# Immediate reduction in left ventricular ejection time following TAVI is associated with improved quality of life

**DOI:** 10.3389/fcvm.2022.988840

**Published:** 2022-09-16

**Authors:** Jimmy Schenk, Eline Kho, Santino Rellum, Joris Kromhout, Alexander P. J. Vlaar, Jan Baan, Martijn S. van Mourik, Harald T. Jorstad, Björn J. P. van der Ster, Berend E. Westerhof, Steffen Bruns, Rogier V. Immink, Marije M. Vis, Denise P. Veelo

**Affiliations:** ^1^Department of Anaesthesiology, Amsterdam UMC, University of Amsterdam, Amsterdam Cardiovascular Sciences, Amsterdam, Netherlands; ^2^Department of Epidemiology and Data Science, Amsterdam UMC, University of Amsterdam, Amsterdam, Netherlands; ^3^Department of Intensive Care, Amsterdam UMC, University of Amsterdam, Amsterdam Cardiovascular Sciences, Amsterdam, Netherlands; ^4^Department of Cardiology, Amsterdam UMC, University of Amsterdam, Amsterdam Cardiovascular Sciences, Amsterdam, Netherlands; ^5^Department of Pulmonary Medicine, Amsterdam UMC, Vrije Universiteit Amsterdam, Amsterdam Cardiovascular Sciences, Amsterdam, Netherlands; ^6^Department of Perinatology, Radboud University Medical Centre, Radboud Institute for Health Sciences, Amalia Children's Hospital, Nijmegen, Netherlands; ^7^Department of Biomedical Engineering and Physics, Amsterdam UMC, University of Amsterdam, Amsterdam Cardiovascular Sciences, Amsterdam, Netherlands

**Keywords:** TAVI, quality of life, ejection fraction, cardiac power index, hemodynamics

## Abstract

**Background:**

TAVI has shown to result in immediate and sustained hemodynamic alterations and improvement in health-related quality of life (HRQoL), but previous studies have been suboptimal to predict who might benefit from TAVI. The relationship between immediate hemodynamic changes and outcome has not been studied before. This study sought to assess whether an immediate hemodynamic change, reflecting myocardial contractile reserve, following TAVI is associated with improved HRQoL. Furthermore, it assessed whether pre-procedural cardiac power index (CPI) and left ventricular ejection fraction (LVEF) could predict these changes.

**Methods:**

During the TAVI procedure, blood pressure and systemic hemodynamics were prospectively collected with a Nexfin^®^ non-invasive monitor. HRQoL was evaluated pre-procedurally and 12 weeks after the procedure, using the EQ-5D-5L classification tool.

**Results:**

Overall, 97/114 (85%) of the included patients were eligible for analyses. Systolic, diastolic and mean arterial pressure, heart rate, and stroke volume increased immediately after TAVI (all *p* < 0.005), and left ventricular ejection time (LVET) immediately decreased with 10 ms (95%CI = −4 to −16, *p* < 0.001). Overall HRQoL_index_ increased from 0.810 [0.662–0.914] before to 0.887 [0.718–0.953] after TAVI (*p* = 0.016). An immediate decrease in LVET was associated with an increase in HRQoL_index_ (0.02 index points per 10 ms LVET decrease, *p* = 0.041). Pre-procedural CPI and LVEF did not predict hemodynamic changes or change in HRQoL.

**Conclusion:**

TAVI resulted in an immediate hemodynamic response and increase in HRQoL. Immediate reduction in LVET, suggesting unloading of the ventricle, was associated with an increase in HRQoL, but neither pre-procedural CPI nor LVEF predicted these changes.

**Clinical trial registration:**

https://clinicaltrials.gov/ct2/show/NCT03088787

## Introduction

The prevalence of severe aortic stenosis (AoS) in elderly (>75 years) is 3.4%, with a yearly mortality rate of 25% (1, 2). Transcatheter aortic valve implantation (TAVI) has been shown to reduce mortality and AoS related symptoms and to improve quality of life in the majority of patients (3). However, the risk of poor outcome 1 year after the procedure varies between 11 and 26% (3). Risk stratifying models, based on patient characteristics such as AoS severity, multi-morbidity, frailty and cognition, have been shown suboptimal in predicting clinical benefit from TAVI (3–5).

Repairing an aortic valve outflow obstruction results in significant hemodynamic alterations. Both immediate and sustained changes following TAVI have been studied, showing an overall increase in systolic blood pressure (6–11) and some (6, 7), but not all (8, 11), found an increase in stroke volume and cardiac output. An increase in blood pressure in the days or even weeks following TAVI has been associated with improved clinical outcome (11–13). However, the relationship between immediate hemodynamic changes and outcome has not been studied.

It has been hypothesized that a baseline difference in myocardial contractile reserve could affect hypertension onset and, consequently, the prognosis following TAVI (11). In addition, a meta-analysis showed an increased risk of mortality in patients with low (<30%) left ventricular ejection fraction (LVEF) compared to patients with normal LVEF (14). A recent study, using the LVEF (<50%) to classify left ventricular dysfunction, did not confirm the previously mentioned hypothesis (15).

Since myocardial contractility and the severity of the AoS can independently vary within and between patients, the averaged fraction of volume ejected by the heart might not be an ideal variable to classify left ventricular dysfunction in this particular population. The cardiac power index (CPI) is the product of simultaneously measured cardiac output and mean arterial pressure, indexed to the body surface area, representing the hydraulic function of the heart (16). CPI has been shown to correlate with varying outcomes in differing populations of patients with cardiovascular disease (17–21). Furthermore, baseline CPI was recently shown to be a strong predictor of 1 year mortality following TAVI (22).

In this study we hypothesize that an immediate hemodynamic response, reflecting a change in myocardial contractility (i.e., contractile reserve) following TAVI is associated with a post-procedural change in health-related quality of life (HRQoL). Furthermore, we aim to assess whether baseline LVEF and CPI, can be used to predict both the immediate hemodynamic response and a change in HRQoL.

## Methods

### Study design and ethical considerations

This was a single center, prospective cohort study conducted at the Amsterdam University Medical Centre, location AMC, in Amsterdam, the Netherlands. Prior to the study, the local medical ethical committee approved the study protocol and the trial was registered with the NIH, U.S. National Library of Medicine at ClinicalTrials.gov (NCT03088787). The trial was conducted in accordance with the ICH Harmonised Tripartite Guideline for Good Clinical Practice, and written informed consent was obtained from each patient prior to inclusion. Patients were recruited on the day prior to their intervention, from the 30th of March 2017 until the 28th of February 2019.

### Study participants

Patients ≥18 years old with severe degenerative aortic valve stenosis, scheduled for TAVI via femoral approach were eligible for inclusion. Patients with a congenital unicuspid or bicuspid valve; being treated with an intra-aortic balloon pump; with an inability to perform a Nexfin measurement at the left-hand side, or a bodyweight below 40 kg were excluded.

### Study outcomes and definitions

The primary outcome of this study was the association of immediate hemodynamic alterations with a change in HRQoL following TAVI. Secondary outcomes were the association of baseline LVEF and CPI with hemodynamic alterations and change in HRQoL.

The studied hemodynamic variables were computed from the continuous blood pressure waveform that was collected using a Nexfin^®^ non-invasive blood pressure monitor at all time-points. Studied variables, at baseline, pre-procedure and post-procedure were: systolic, diastolic, and mean arterial pressure (MAP, mmHg); heart rate (beats·min^−1^); stroke volume (SV, ml); cardiac output (CO, L·min^−1^); systemic vascular resistance (SVR, dynes·s·cm^−5^); left ventricular ejection time (LVET, ms), and the maximal rate of rise of systolic pressure (dP/dt, mmHg·s^−1^). SV was calculated with the ^cc^Nexfin CO-Trek algorithm, dividing the time-integral area under the systolic part of the arterial pressure curve by the aortic input impedance (23–25). CO was then calculated by multiplying SV with heart rate. Stroke work (SW) was calculated as SV multiplied by MAP (ml·mmHg^−1^).

The hydraulic function of the heart was defined as the CPI (W·m^2^) and was calculated as [(MAP ^*^ CO/451)] / body surface area (BSA, m^2^) (19). CPI was additionally classified as low (<0.44 W·m^−2^) or normal (≧0.44 W·m^−2^) according to results by Grodin et al. (21). The EQ-5D-5L health state classification (26) was used to evaluate HRQoL.

### Study procedures

Patients were treated according to the TAVI-procedure standard of practice, were kept awake and received local anesthesia. All patients received an Edwards SAPIEN 3 Transcatheter Valve, with some patients requiring aortic valvuloplasty prior to valve implementation.

### Data collection and analyses

Baseline characteristics, including medical history, and transthoracic echocardiogram (TTE) findings were collected from electronic patient records. Pre-procedural left ventricular function (LVF) grade was collected from TTE findings. Pre-procedural LVEF was determined using automatic whole-heart segmentation in 4D Coronary Computed Tomography Angiography (CCTA). This deep learning-based method segments the cardiac chambers and myocardium, allowing automatic identification of end-systolic and end-diastolic phases and subsequent calculation of the ejection fraction (27). HRQoL status was evaluated pre-procedurally in the hospital, and repeated 12 weeks after the procedure by phone. HRQoL_index_ scores were calculated using the Dutch tariff value set (28), ranging from −0.446 to 1, with a negative score indicating a health state worse than death.

Before starting the procedure, a finger cuff with a light-emitting and light sensitive diode for plethysmography (Nexfin, Edwards Lifesciences, Irvine, CA, USA) was strapped around the middle phalanx of the middle or index finger at the left hand to obtain a non-invasive continuous blood pressure registration (sampled at 200 Hz). Measurements were stopped at the end of the procedure, at discharge to a nursing ward.

**Figure 1:**
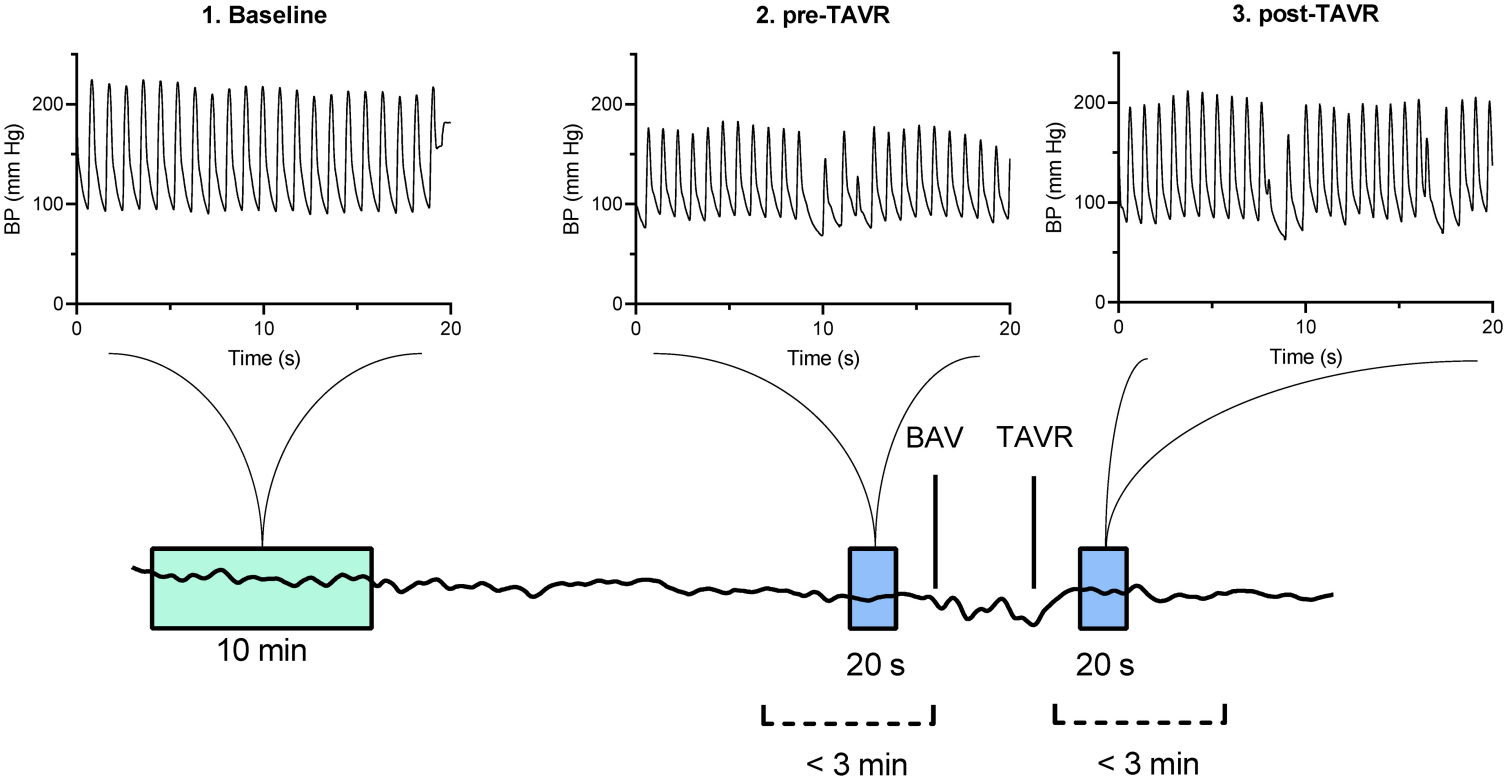
Timeframe and measurement selection, visualized within a random patients' total procedure waveform data. BAV, balloon aortic valvuloplasty; TAVI, Transcatheter Aortic Valve Implantation.

Offline analysis of the blood pressure waveform data was performed with MATLAB (MathWorks, Inc, Natick, MA). Two researchers (JS and EK) manually selected three pre-defined artifact-free time frames. The baseline time frame consisted of 10 min of blood pressure data, collected in the treatment room in a supine position before the start of the procedure. The direct pre-TAVI time frame consisted of 20 s of artifact-free waveform data and was selected in the 3 min of data measured directly before valve implantation, or before initial aortic valvuloplasty, when performed. The direct post-TAVI time frame was selected in the 3 min of data measured directly after valve implantation (Figure 1).

Patients in whom no artifact-free waveform data could be selected, and patients who either needed pacemaker support or showed newly onset arrhythmia in the previously defined time windows, were excluded from further analysis.

### Sample size

The sample size calculation technique for multiple regression, as defined by Green (29), was used to calculate the sample size. The effect size (f^2^) of TAVI on the average HRQoL_index_ score was estimated at 0.2. Given the a-priori interest in the association of ten predictors, 91 patients would provide 80% power to detect a statistically significant association for each predictor, with a 0.05 two sided significance level.

### Statistical analyses

Continuous data are presented as median with interquartile range (IQR), or as a mean with standard deviation (SD) when normally distributed. Normality of distribution was assessed visually using histograms and Q-Q plots. Differences between continuous data were analyzed using the Student's *t*-test when normally distributed, or using the Wilcoxon rank-sum test when non-normally distributed. Categorical data are presented as frequencies with percentages. Differences between categorical data were analyzed using the Fisher's exact test. Differences in the repeated measurements of hemodynamic variables (pre-TAVI vs. post-TAVI) were analyzed using the paired Student's *t*-test. During the planning stage of this study, valvuloplasty was identified as a possible confounding variable. It likely results in an increase in elapsed time between the pre- and post-TAVI measurement and has shown to induce ventricular stunning, potentially affecting pressure measurements (30). Potential group differences in immediate hemodynamic changes between patients with and without valvuloplasty prior to valve implementation were analyzed using generalized linear mixed-effect models.

Furthermore, generalized linear mixed-effect models were used to analyze: the association of immediate hemodynamic changes with change in HRQoL; the association of pre-procedural LVEF and CPI with immediate hemodynamic changes; and the association of pre-procedural CPI with change in HRQoL. Multiple imputation was used to impute missing data, assuming the data to be missing at random, validated by Little's MCAR test (31). When data was deemed missing at random, the multivariate imputation by chained equations (MICE) (32) method was used to impute data. For each of the analyses, *p* < 0.05 was considered statistically significant. Statistics were done using R v4.0.3 (R Core Team, Vienna, Austria), employing the nlme (v3.1-152) and the mice (v3.13.0) packages. JS had full access to all the data in the study and takes responsibility for its integrity and the data analysis.

## Results

**Figure 2:**
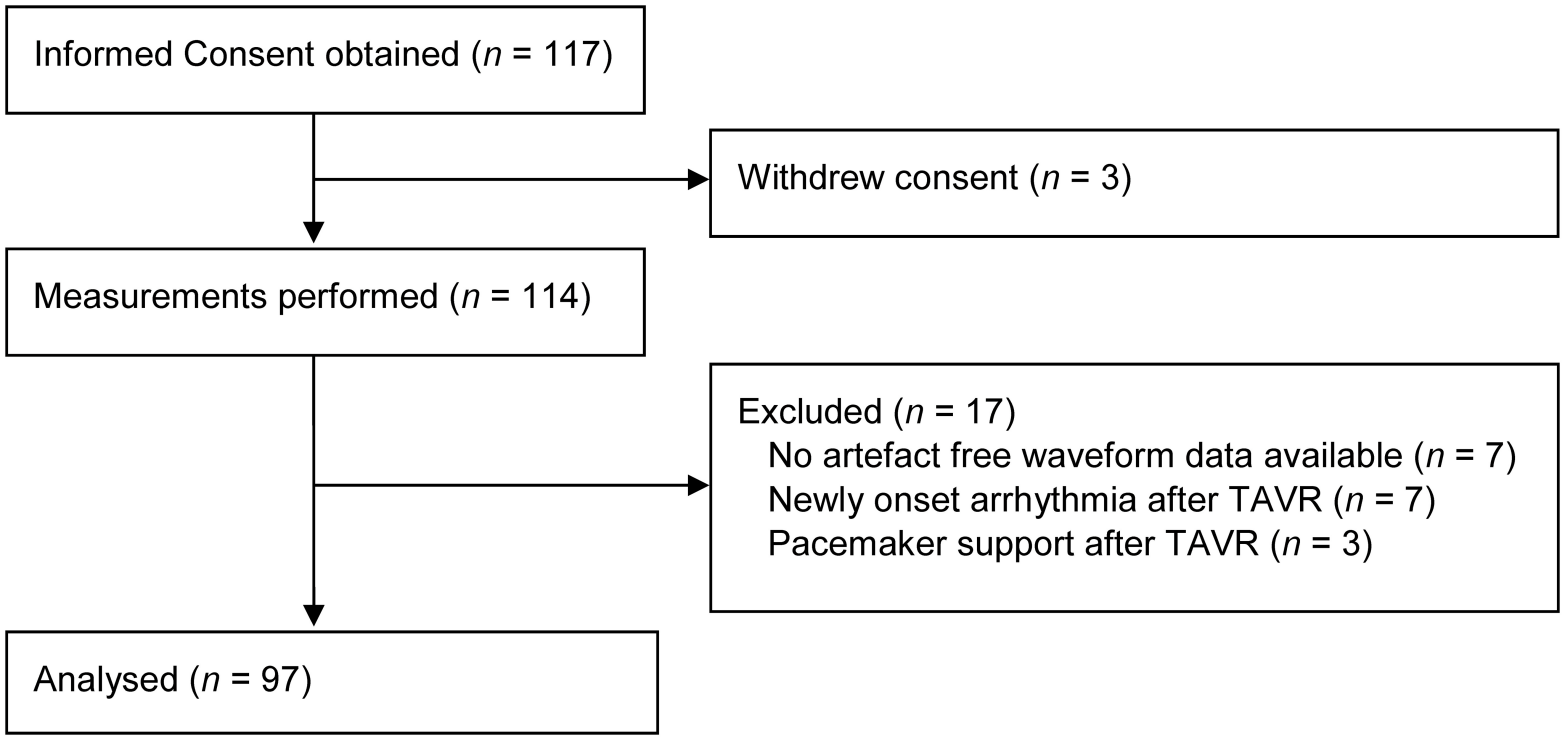
Study flow diagram.

Measurements were performed in 114 patients, of whom 97 were eligible for analysis. No artifact-free waveform data could be selected in seven patients, seven other patients showed newly onset arrhythmia, and three patients were depending on pacemaker support directly following valve implantation (Figure 2). Table 1 shows the baseline characteristics of patients included for analysis. Mean age was 81 ± 6 years, median NT-pro-BNP level was 1,173 pg·ml^−1^ [581–3,121], and most patients suffered from pre-existing hypertension (60%). Table 2 shows the averages of pre-procedural TTE measurements. Aortic stenosis was graded as severe in most patients (92%), with a mean aortic valve area of 0.78 ± 0.18 cm^2^. The average aortic valve mean,- and maximum gradient were 38.8 ± 15.4 mmHg and 65.5 ± 24.3 mmHg, respectively. The average pre-procedural LVEF was calculated at 54 ± 17%, and showed agreement with the TTE graded left ventricular function (Supplementary Figure 1).

**Table 1:** Baseline characteristics.

	**Overall**
	**(*n =* 97)**
Male (%)	43 (44.3)
Age (y)	81 (5.6)
Weight (kg)	79.2 (18.3)
Height (cm)	167 (9)
BMI (kg·m^−2^)	28.1 (5.7)
**ASA classification (%)**
I	1 (1.0)
II	6 (6.2)
III	73 (75.3)
IV	17 (17.5)
MET score (median [IQR])	6 [5, 6]
**Medical history (%)**
Hypertension	58 (59.8)
Dyslipidemia	29 (29.9)
DM type II	29 (29.9)
Congestive heart failure	18 (18.6)
CVA	16 (16.5)
Myocardial Infarction	14 (14.4)
COPD	14 (14.4)
None	15 (15.5)
**Pre-procedural hearth rhythm (%)**
Sinus rhythm	69 (71.1)
Atrial fibrillation	19 (19.6)
Other	9 (9.3)
NT-proBNP (median [IQR])	1173 [510, 3121]
LVEF	54 (17)

BMI, body mass index; ASA, American Society of Anaesthesiologists; MET, metabolic equivalent task; DM, diabetes mellitus; CVA, cerebral vascular accident; COPD, chronic obstructive pulmonary disease; NT-proBNP, N-terminal prohormone of brain natriuretic peptide; LVEF, left ventricular ejection fraction.

**Table 2:** Pre-procedural transthoracic echocardiogram results.

	**Overall**
	**(*n =* 97)**
**Left ventricular function grade (%)**
Good	46 (47.9)
Mildly impaired	31 (32.3)
Moderately impaired	10 (10.4)
Poor	7 (7.3)
Very poor	2 (2.1)
Left Ventricular Hypertrophy (%)	58 (65.2)
**Right ventricular function grade (%)**
Good	75 (83.3)
Mildly impaired	9 (10.0)
Moderately impaired	5 (5.6)
Poor	1 (1.1)
Very poor	0
**Aortic insufficiency grade (%)**
None	16 (18.4)
Trace	9 (10.3)
Grade 1: Mild	44 (50.6)
Grade 2: Moderate	13 (14.9)
Grade 3: Moderate to severe	2 (2.3)
Grade 4: Severe	3 (3.4)
**Aortic stenosis grade (%)**
Mild	2 (2.1)
Moderate	6 (6.4)
Severe	86 (91.5)
Aortic valve area (cm^2^)	0.78 (0.18)
Aortic valve area index (cm^2^/m^2^)	0.38 (0.13)
Aortic valce max gradient (mmHg)	65.48 (24.26)
Aortic valve mean gradient (mmHg)	38.84 (15.38)

Pre-procedural transthoracic echocardiogram results were collected from patient records. Measurements and grading were performed and documented by an echocardiography specialist.

### Immediate hemodynamic changes after TAVI

**Figure 3:**
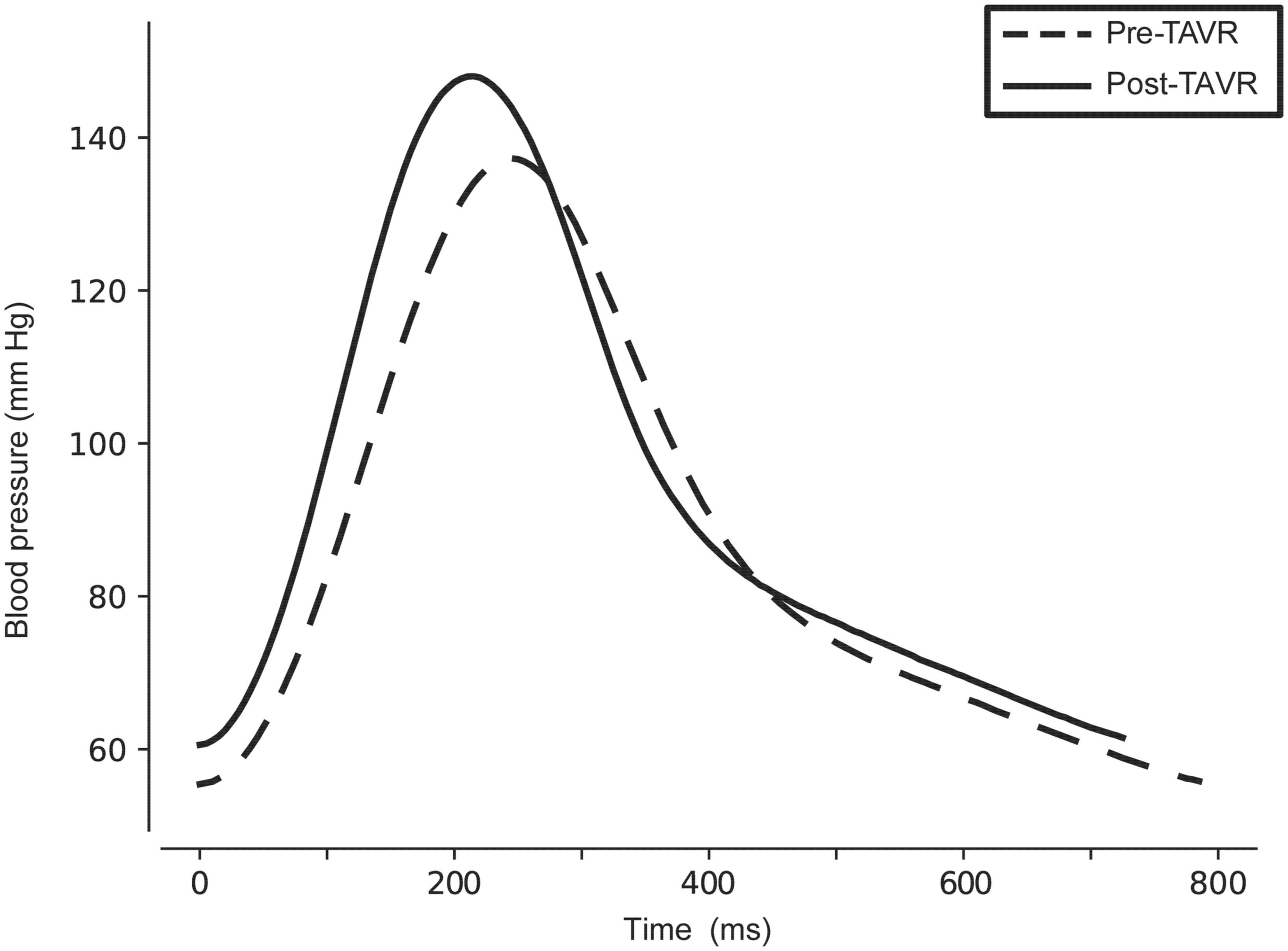
Visualization of the immediate blood pressure waveform change following TAVI. To compose this figure, waveform data of a representative patient was used, whose changes were in line with the average change of the studied population. Pre- (dashed) and Post-TAVI (solid) waveforms were composed by selecting the first 15 beats of each respective time window, trimmed to have the same duration and finally averaged.

The immediate change in hemodynamic variables was calculated for each patient and then averaged (Table 3, Figure 3). On average, systolic, diastolic and mean arterial pressure increased significantly, as did the average heart rate and stroke volume. Left ventricular ejection time was reduced with 10 ms (95%CI = −4 ms to −16 ms, *p* < 0.001) and the maximal rate of rise of systolic pressure (dP/dt) was increased by 67% (414 mmHg·s^−1^, 95%CI = 335 mmHg·s^−1^-494 mmHg·s^−1^, *p* < 0.001). There was no statistically significant immediate change in systemic vascular resistance.

**Table 3:** Immediate hemodynamic changes following TAVI.

	**Pre-TAVI (SD)**	**Post-TAVI (SD)**	**Immediate change (95% CI)**	**% change**	***p*-value**
Systolic pressure (mmHg)	137.5 (27)	151.8 (31.9)	14.2 (9.4 to 19.0)	11%	**<0.001**
Diastolic pressure (mmHg)	65.6 (11.2)	68.9 (12.3)	3.2 (1.3 to 5.2)	6%	**0.001**
MAP (mmHg)	91.4 (15.8)	98 (18)	6.6 (3.7 to 9.5)	8%	**<0.001**
HR (beats·min^−1^)	72.7 (15.5)	76.2 (15.1)	3.6 (1.3 to 5.8)	7%	**0.002**
SV (ml)	69.2 (21.2)	72.6 (20.4)	3.4 (1.4 to 5.4)	7%	**0.001**
CO (L·min^−1^)	4.9 (1.7)	5.4 (1.8)	0.5 (0.3 to 0.7)	14%	**<0.001**
SVR (dynes·sec·cm^−5^)	1675 (722)	1604 (661)	−71 (−165 to 24)	0%	0.142
LVET (ms)	332 (33)	322 (33)	−10 (−16 to −4)	−3%	**<0.001**
dP/dt (mmHg·sec^−1^)	724 (368)	1138 (575)	414 (335 to 494)	67%	**<0.001**
SW (ml·mmHg^−1^)	6349 (2210)	7071 (2358)	722 (517 to 927)	14%	**<0.001**

Pre-TAVI and Post-TAVI values are given as mean with standard deviation. MAP, mean arterial pressure; HR, heart rate; SV, stroke volume; CO, cardiac output; SVR, systemic vascular resistance; LVET, left ventricular ejection time; dP/dt, maximal rate of rise of systolic pressure; SW, stroke work. Bold values indicate a statistically significant difference between the Pre-TAVI and Post-TAVI value.

There was a 336 s difference (95%CI = 269 s−402 s, *p* < 0.001) in elapsed time between pre-, and post-TAVI measurements when comparing patients with and without aortic valvuloplasty. When comparing these groups, no significant differences in immediate hemodynamic response were found.

### Primary outcome: The association of immediate hemodynamic changes with changes in health-related quality of life

Median baseline HRQoL_index_ score was 0.810 [0.662–0.914], and increased to 0.887 [0.718–0.953] after the procedure (Wilcoxon rank sum, *p* = 0.016). Baseline characteristics of patients with stable or improved HRQoL (*n* = 64) were comparable with those of patients with decreased HRQoL (*n* = 33; Supplementary Tables 1, 2). The post-procedure index score of one patient and baseline index scores of nine patients were imputed.

**Figure 4:**
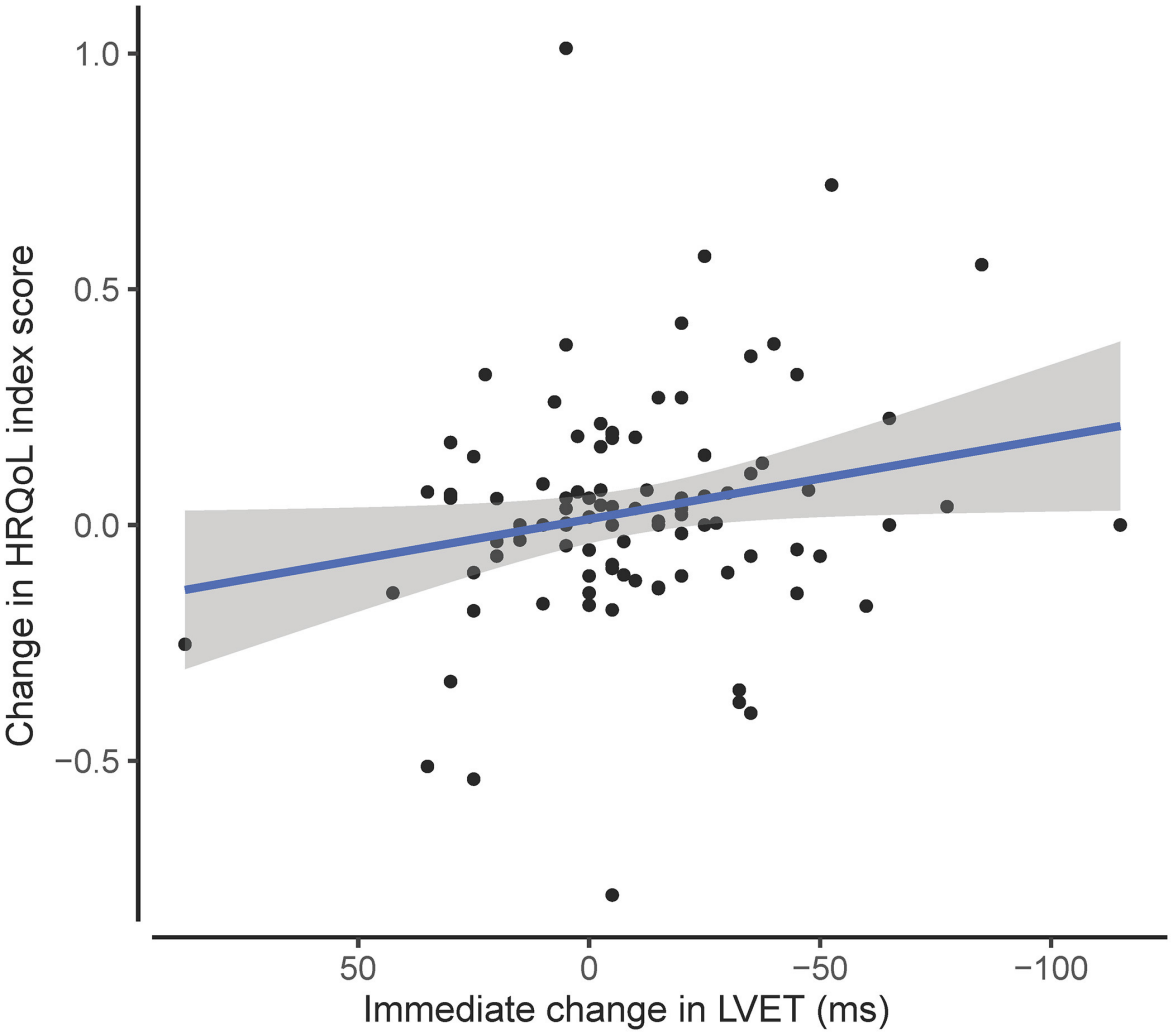
The association of the immediate change in LVET with the change in HRQoL index score following TAVI. Post TAVI HRQoL index score increases with 0.02 index points per 10ms immediate decrease in LVET (*p* = 0.042). HRQoL, health related quality of life; LVET, left ventricular ejection time.

Employing generalized linear mixed models, and corrected for within-subject correlation in repeated measures, an immediate decrease in LVET was associated with a post-procedural increase in HRQoL_index_ (0.02 index points increase per 10 ms LVET decrease, *p* = 0.041; Figure 4). Immediate changes in pressure (systolic, diastolic and mean arterial) and changes in other hemodynamic variables were not associated with a change in HRQoL.

### Secondary outcomes

Median baseline CPI was 0.50 [0.38–0.64], with CPI classified as low in 36 patients and normal in 60 patients. Normal baseline CPI predicted a higher immediate change in SV (3.97 ml difference, *p* = 0.049) and a larger immediate change in SVR (240 dynes·sec·cm^−5^ difference, *p* = 0.015). Baseline CPI classifications did not predict a change in HRQoL. There was no association of pre-procedural LVEF with any of the hemodynamic changes or change in HRQoL.

## Discussion

In this study we hypothesized that an immediate hemodynamic response, reflecting a change in myocardial contractility (i.e., contractile reserve), following TAVI would be associated with a post-procedural change in HRQoL. We confirm that TAVI resulted in an overall significant increase in HRQoL and found that an immediate decrease in LVET was associated with an increase in HRQoL. We confirmed the immediate hemodynamic response found in previous research (6–9), showing an increase in blood pressure, stroke volume, cardiac output and maximal rate of rise of systolic pressure (dP/dt) accompanied by a decrease in LVET, without a significant change in systemic vascular resistance. The secondary aim of this study was to investigate whether the hemodynamic and HRQoL changes could pre-procedurally be predicted using either the LVEF or CPI. We found that pre-procedural LVEF was not associated with any of the changes, and that the CPI could predict the amount of immediate change in stroke volume and vascular resistance, but was not prognostic for change in HRQoL.

TAVI resulted in a significant and clinically relevant improvement in HRQoL, which is in line with various other studies (33–38). Patients requiring TAVI are mostly elderly with a high surgical risk, reduced exercise capacity, fatigue, and as a result, reduced HRQoL. Rather than mortality, improvement in quality of life is the most important patient-related outcome following TAVI (33). While association does not imply causation, the reduction in LVET found in this study might reflect the adaptive capacity of the left ventricle, following the sudden repair of the aortic outflow obstruction. We hypothesize that, when left ventricular volume loading remains equal and the afterload is greatly and suddenly reduced, an immediate reduction in time and contractile effort needed to eject the volume can be expected in patients with normal left ventricular function. Consequently, a reduction in LVET might reflect a myocardial contractile adaptive capacity, rather than a myocardial contractile reserve, previously hypothesized as the underlying mechanism for a difference in improved outcome following TAVI (11). Thus, we hypothesize that the immediate increase in maximal rate of rise of systolic pressure (dP/dt) simply reflects the sudden afterload reduction, rather than an increase in left ventricular contractility. When the left ventricle has the capacity to immediately adapt to the TAVI induced afterload reduction, pre-procedural symptoms that are primarily caused by the outflow obstruction might reduce, which could explain a potential increase in HRQoL. Patients with reduced adaptive capacity might show less immediate changes in hemodynamic variables reflecting this capacity, despite reduction of the outflow obstruction. Consequently, these patients might show less reduction of pre-procedural symptoms and, with it, less change in the quality of life following the procedure.

Various studies have shown that an increase in heart rate can affect the LVET in a sample of patients without alterations to their cardiac structure. However, studies showing the linear relationship of heart rate with ejection time were conducted in a steady-state of circulating volume. We believe that, in this very specific sample, adjusting for the increase in heart rate would result in overcorrection and might induce a type II error. This is underlined by the fact that, besides an average 7% increase in HR, there also was an average 7% increase in stroke volume while LVET still decreased. The removal of the aortic stenosis thus allows the left ventricle to eject more volume, in a smaller amount of time. Moreover, when we analyzed the relationship between LVET and quality of life index in a multivariable regression and the change in heartrate (corrected for the change in stroke volume) is added as an effect modifying factor, the regression coefficient is altered by <10%, indicating that there is no significant effect modification (Supplementary Table 3).

The immediate reduction in LVET might already be present after initial valvuloplasty, which we did not assess in this study. It would be interesting for future studies to measure whether the subsequent valve implementation would have any additional hemodynamic effect in patients that did not immediately show LVET reduction after valvuloplasty. When this is not the case, the immediate hemodynamic response after initial valvuloplasty might be indicative of the additional therapeutic effect of a subsequent valve implementation. This question was beyond the scope of this study. Furthermore, future studies might be able to provide insight in the correlation of immediate LVET reduction following TAVI with sustained increased blood pressure and mortality.

We found no relevant differences in baseline characteristics between patients with reduced HRQoL and patients with stable or improved HRQoL. Even though severely reduced pre-procedural LVEF has shown to predict mortality after TAVI (14), LVEF was not associated with differences in immediate hemodynamic alterations or change in HRQoL in our study. The CPI has previously shown to be the strongest hemodynamic correlate of mortality in varying cardiac patient groups (20), including the TAVI population (22). We hypothesized that the pre-procedural CPI would allow prediction of immediate hemodynamic alterations and improvement in HRQoL. Our results show that CPI, in line with LVEF, was unable to pre-procedurally identify patients that show an increase in HRQoL following TAVI. Furthermore, CPI was unable to predict the immediate alterations in variables reflecting a change in myocardial contractility.

### Limitations

It has previously been shown that rapid ventricular pacing used for aortic valvuloplasty and valve deployment can result in ventricular stunning, which could have affected pressure measurements (30). However, when the immediate hemodynamic responses between patients with and without aortic valvuloplasty were compared, no significant differences were found, indicating that the potential additional impact of ventricular stunning in patients requiring valvuloplasty did not alter the results.

The cohort consisted of mainly elderly patients with severe AS. Therefore, the results might not be generalizable to other patient groups. Furthermore, the sample size of this prospectively collected cohort was not large, but the hemodynamic changes and the associations found were highly significant, indicating validity of the results. Additionally, since no flow data was collected, stroke volume and consequently the cardiac output were calculated with the ^cc^Nexfin CO-Trek algorithm. Employing this algorithm to calculate stroke volume has shown to be less precise in specific subgroups of critically ill patients (39, 40). Non-invasive continuous blood pressure measurement has shown to be accurate in patients with severe aortic stenosis (41, 42). It is unclear whether severe aortic stenosis could affect stroke volume estimations. Since our calculations were based on repeated measurement within each patient, the percentile change in stroke volume is likely to accurately reflect the alterations following TAVI. The accuracy of non-invasively measuring change in stroke volume is underlined by comparable findings, where invasively acquired pressure waveforms in the ascending aorta were used to assess the acute hemodynamic effects following TAVI, using an identical methodology in time frame selection (8).

The increase in HRQoL_index_ score was considered a clinically relevant change. The increase was larger than the estimation of minimally important difference when using the EQ-5D-5L health state classification tool, ranging from 0.037 to 0.069 (43). However, even though the EQ-5D-5L tool is easy to use and understand, it might pose a limitation due to its potential ceiling effect (26). Comparable future studies could provide additional insight using a more extensive survey, such as the Medical Outcomes Study 36-Item Short Form (44).

## Conclusions

TAVI resulted in an immediate hemodynamic response and an increase in HRQoL. Immediate reduction in LVET, suggesting unloading of the ventricle, was associated with an increase in HRQoL, but neither the pre-procedural CPI nor LVEF was able to predict these changes.

## Data availability statement

The original contributions presented in the study are included in the article/Supplementary material, further inquiries can be directed to j.schenk@amsterdamumc.nl.

## Ethics statement

The studies involving human participants were reviewed and approved by Medisch Ethische Toetsingscommissie (METC): Amsterdam University Medical Centre, location AMC, Amsterdam, the Netherlands. The patients/participants provided their written informed consent to participate in this study.

## Author contributions

JS: conception, design, analysis and interpretation of data, and drafting of the manuscript. EK: analysis and interpretation of data and final approval of the manuscript submitted. SR and MV: conception and design and final approval of the manuscript submitted. JK, AV, JB, BW, and SB: final approval of the manuscript submitted. MM and HJ: conception and final approval of the manuscript submitted. BS: interpretation of data and final approval of the manuscript submitted. RI: design and final approval of the manuscript submitted. DV: conception, design, interpretation of data, and final approval of the manuscript submitted. All authors contributed to the article and approved the submitted version.

## Conflict of interest

Author AV has received personal fees and other from Edwards Lifesciences and Philips outside the submitted work. Author RI has received a grant from Edwards Lifesciences outside the submitted work. Author JB has received a grant from Edwards Lifesciences outside the submitted work. Author DV has received personal fees and other from Edwards Lifesciences, Philips and Hemologic outside the submitted work. The remaining authors declare that the research was conducted in the absence of any commercial or financial relationships that could be construed as a potential conflict of interest.

## Publisher's note

All claims expressed in this article are solely those of the authors and do not necessarily represent those of their affiliated organizations, or those of the publisher, the editors and the reviewers. Any product that may be evaluated in this article, or claim that may be made by its manufacturer, is not guaranteed or endorsed by the publisher.

## Abbreviations

AoS: aortic stenosis
CPI: cardiac power index
HRQoL: health related quality of life
LVEF: left ventricular ejection fraction
LVET: left ventricular ejection time
TAVI: transcatheter aortic valve implantation
TTE: transthoracic echocardiogram.
