# Supplementary material for: Immediate reduction in left ventricular ejection time following TAVI is associated with improved quality of life
Source: Front Cardiovasc Med. 2022 Sep 16;9:988840. doi: 10.3389/fcvm.2022.988840 (PMC9523106; doi:10.3389/fcvm.2022.988840)
Supplement: Supplementary file 1 [file Data_Sheet_1.docx]

# *Supplementary material*


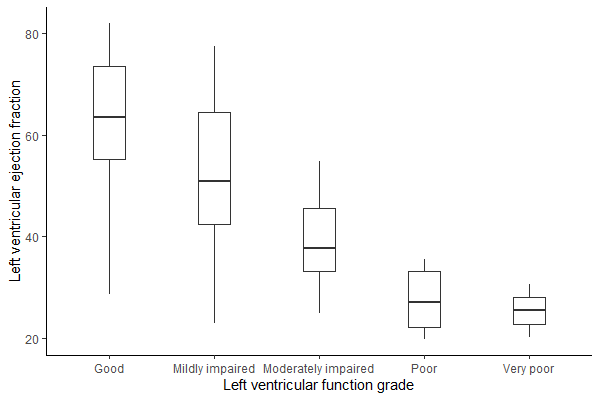


**Supplementary figure 1.** Agreement between left ventricular ejection fraction, derived from automatic whole-heart segmentation in 4D Coronary Computed Tomography Angiography, and transthoracic echocardiogram left ventricular function grade.

| **Supplementary table 1.**  Differences in baseline characteristics between stable or improved and decreased HRQoL | | | |
| --- | --- | --- | --- |
|  | HRQoL stable or improved | HRQoL decreased | *p*-value |
|  | n=64 | n=33 |  |
| Male (%) | 32 (50) | 11 (33) | 0.177 |
| Age (y) | 80 (6) | 82 (5) | 0.058 |
| Weight (kg) | 81 (19) | 76 (16) | 0.198 |
| Height (cm) | 168 (10) | 166 (8) | 0.335 |
| BMI (kg⋅m^-2^) | 28.5 (5.8) | 27.4 (5.5) | 0.346 |
| Medical History (%) |  |  |  |
| Hypertension | 39 (60.9) | 19 (57.6) | 0.919 |
| Dyslipidemia | 22 (34.4) | 7 (21.2) | 0.268 |
| DM type II | 22 (34.4) | 7 (21.2) | 0.268 |
| Congestive heart failure | 15 (23.4) | 3 (9.1) | 0.148 |
| CVA | 9 (14.1) | 7 (21.2) | 0.542 |
| Myocardial Infarction | 11 (17.2) | 3 (9.1) | 0.441 |
| COPD | 10 (15.6) | 4 (12.1) | 0.873 |
| None | 9 (14.1) | 6 (18.2) | 0.814 |
| Pre-procedural Hearth rhythm (%) |  |  | 0.684 |
| Sinus rhythm | 44 (68.8) | 25 (75.8) |  |
| Atrial fibrillation | 13 (20.3) | 6 (18.2) |  |
| Other | 7 (10.9) | 2 (6.1) |  |
| NT-proBNP (median [IQR]) | 1104 [546, 2982] | 1400 [712, 3453] | 0.563 |
| LVEF (mean (SD)) | 53 (16) | 57 (18) | 0.307 |

BMI = body mass index, ASA = American Society of Anesthesiologists, MET = metabolic equivalent task, DM = diabetes mellitus, CVA = cerebral vascular accident, COPD = chronic obstructive pulmonary disease, NT-proBNP = N-terminal prohormone of brain natriuretic peptide, LVEF = left ventricular ejection fraction.

| **Supplementary table 2** | | |  | |  | | | |
| --- | --- | --- | --- | --- | --- | --- | --- | --- |
| Differences in pre-procedural transthoracic echocardiogram results between stable or improved and decreased HRQoL | | | | | | |  |  |
|  | | HRQoL stable or improved | | HRQoL decreased | | *p*-value | | |
|  | | n=64 | | n=33 | |  | | |
| Left Ventricular Function grade (%) | |  | |  | | 0.082 | | |
| Good | 26 (41.3) | | 20 (60.6) | |  | | |  |
| Mildly impaired | 21 (33.3) | | 10 (30.3) | |  | | |  |
| Moderately impaired | 10 (15.9) | | 0 (0.0) | |  | | |  |
| Poor | 4 (6.3) | | 3 (9.1) | |  | | |  |
| Very poor | 2 (3.2) | | 0 (0.0) | |  | | |  |
| Left Ventricular Hypertrophy (%) | | 33 (57.9) | | 25 (78.1) | | 0.091 | | |
| Right Ventricular Function grade (%) | |  | |  | | 0.602 | | |
| Good | 47 (79.7) | | 28 (90.3) | |  | | |  |
| Mildly impaired | 7 (11.9) | | 2 (6.5) | |  | | |  |
| Moderately impaired | 4 (6.8) | | 1 (3.2) | |  | | |  |
| Poor | 1 (1.7) | | 0 (0.0) | |  | | |  |
| Very poor | 0 (0.0) | | 0 (0.0) | |  | | |  |
| Aortic insufficiency grade (%) | |  | |  | | 0.786 | | |
| None | 11 (18.6) | | 5 (17.9) | |  | | |  |
| Trace | 7 (11.9) | | 2 (7.1) | |  | | |  |
| Grade 1: Mild | 29 (49.2) | | 15 (53.6) | |  | | |  |
| Grade 2: Moderate | 8 (13.6) | | 5 (17.9) | |  | | |  |
| Grade 3: Moderate to severe | 1 (1.7) | | 1 (3.6) | |  | | |  |
| Grade 4: Severe | 3 (5.1) | | 0 (0.0) | |  | | |  |
| Aortic stenosis grade (%) | |  | |  | | 0.396 | | |
| Mild | 2 ( 3.1) | | 0 ( 0.0) | |  | | |  |
| Moderate | 3 ( 4.7) | | 3 (10.0) | |  | | |  |
| Severe | 59 (92.2) | | 27 (90.0) | |  | | |  |
| Aortic Valve Area (cm²) | | 0.80 (0.19) | | 0.75 (0.15) | | 0.250 | | |
| Aortic Valve Area index (cm²/m^2^) | | 0.38 (0.15) | | 0.39 (0.06) | | 0.830 | | |
| Aortic Valce max gradient (mm Hg) | | 65.82 (23.47) | | 64.78 (26.18) | | 0.849 | | |
| Aortic Valve mean gradient (mm Hg) | | 38.14 (13.40) | | 40.00 (18.42) | | 0.621 | | |
|  | | |  | |  | | | |

| **Supplementary table 3** |  |  | |
| --- | --- | --- | --- |
| Regression model output for the unadjusted and adjusted association of immediate difference in LVET with change in HRQoL | | |  |
| ***Unadjusted association*** | | |  |
| \| *Predictors* \| *Estimates* \| *std. Error* \| *CI* \| *Statistic* \| *p* \| \| --- \| --- \| --- \| --- \| --- \| --- \| \| (Intercept) \| 0.1647 \| 0.2783 \| -0.3821 – 0.7114 \| 0.59 \| 0.555 \| \| Δ LVET (per 10ms decrease) \| 0.0183 \| 0.0089 \| 0.0009 – 0.0357 \| 2.06 \| **0.042** \| \| ***Adjusted association*** \| \| \| \| \| \| \| (Intercept) \| 0.2742 \| 0.2813 \| -0.2754 – 0.8239 \| 0.98 \| 0.332 \| \| Δ LVET (per 10ms decrease) \| 0.0171 \| 0.0091 \| -0.0006 – 0.0348 \| 1.89 \| 0.062 \| \| Δ HR_corrected_ \| 0.0343 \| 0.0617 \| -0.0863 – 0.1549 \| 0.56 \| 0.580 \| | | |  |
